# Prediction and functional analysis of the sweet orange protein-protein interaction network

**DOI:** 10.1186/s12870-014-0213-7

**Published:** 2014-08-05

**Authors:** Yu-Duan Ding, Ji-Wei Chang, Jing Guo, DiJun Chen, Sen Li, Qiang Xu, Xiu-Xin Deng, Yun-Jiang Cheng, Ling-Ling Chen

**Affiliations:** 1 Key Laboratory of Horticultural Plant Biology of Ministry of Education, Huazhong Agricultural University, Wuhan 430070, People’s Republic of China; 2 Agricultural Bioinformatics Key laboratory of Hubei Province, College of Information, Huazhong Agricultural University, Wuhan 430070, People’s Republic of China

**Keywords:** Protein-protein interaction, Ortholog, Domain, Modular, Plant hormone

## Abstract

**Background:**

Sweet orange (*Citrus sinensis*) is one of the most important fruits world-wide. Because it is a woody plant with a long growth cycle, genetic studies of sweet orange are lagging behind those of other species.

**Results:**

In this analysis, we employed ortholog identification and domain combination methods to predict the protein-protein interaction (PPI) network for sweet orange. The K-nearest neighbors (KNN) classification method was used to verify and filter the network. The final predicted PPI network, CitrusNet, contained 8,195 proteins with 124,491 interactions. The quality of CitrusNet was evaluated using gene ontology (GO) and Mapman annotations, which confirmed the reliability of the network. In addition, we calculated the expression difference of interacting genes (*EDI*) in CitrusNet using RNA-seq data from four sweet orange tissues, and also analyzed the *EDI* distribution and variation in different sub-networks.

**Conclusions:**

Gene expression in CitrusNet has significant modular features. Target of rapamycin (TOR) protein served as the central node of the hormone-signaling sub-network. All evidence supported the idea that TOR can integrate various hormone signals and affect plant growth. CitrusNet provides valuable resources for the study of biological functions in sweet orange.

## Background

Protein-protein interactions (PPIs) are involved in almost all aspects of cellular processes. Understanding the interactions between proteins is an important goal of systems biology, and such knowledge can provide crucial insights into protein function and molecular mechanisms. Various experimental technologies, such as affinity purification mass spectrometry (AP-MS) [1], the yeast two-hybrid (Y2H) system [2], and protein arrays [3]–[5] have been applied to detection of genome-wide PPIs in many model species, including *Homo sapiens*[6], *Drosophila melanogaster*[7], *Saccharomyces cerevisiae*[8], and *Caenorhabditis elegans*[9]. However, technical problems limit the efficacy of some of the aforementioned high-throughput screening methods. For example, the PPIs identified to date represent only a small fraction of the full PPI network in the relevant species. Furthermore, the techniques used to identify PPIs are labor-intensive and costly, further limiting the application of these methods. In addition, most experimental methods have condition- or method-specific features; consequently, the data obtained by various methods sometimes exhibit minimal overlap even within the same species.

To overcome the disadvantages of existing experimental methods and expand the coverage of PPIs, various bioinformatics approaches have been developed, including ortholog- and domain-based methods, gene neighbor methods, and gene fusion methods [10]–[16]. Various types of data are used to predict PPIs, including protein sequences and functions, protein structures, gene expression, *etc*. Many public databases have been constructed to store the experimental and theoretical predicted PPIs, including the Database of Interacting Proteins (DIP) [17], Molecular INTeraction database (MINT) [18], protein InterAction database (IntAct) [19], Search Tool for the Retrieval of Interacting Genes/Proteins (STRING) [20], Biological General Repository for Interaction Datasets (BioGRID) [21], and the Human Protein Reference Database (HPRD) [22].

Currently, the comprehensive determination of protein interactomes in plant species is lagging behind analogous efforts in model animals. Using Y2H, Dreze and colleagues generated a highly reliable binary PPI network of *Arabidopsis thaliana* containing about 6,200 interactions among 2,700 proteins [23]. To date, computational prediction of PPIs has only focused on a few model plant species, such as *A. thaliana*[24]–[26] and *Oryza sativa*[27]. Research on protein interactomes in perennial woody plants is lacking, in part due to limited genetic studies in these species.

*Citrus* is commercially cultivated in over 130 countries and districts, and citrus fruit holds a dominant position in the global fruit industry (http://faostat.fao.org). Although many germplasms and cultivars exist, comprehensive morphological, anatomical, and molecular evidences has confirmed that all modern cultivars (such as sweet orange) are the derived offspring of two primitive species, pummel (*C. grandis*) and mandarin (*C. reticulata*) [28],[29]. Genomic evidence, including a large body of genetic information from *Citrus*, has demonstrated that sweet orange is a natural hybrid of pummel and mandarin [28],[30]. Sweet orange, which is consumed fresh and as juice, contains large amounts of vitamin C and other compounds beneficial to human health; this species accounts for more than 60% of world fruit production (http://faostat.fao.org). Thus, due to its economic and genetic importance, sweet orange is an important woody model plant. The draft genome of this species provides an unprecedented opportunity to investigate its genetics, biochemistry, and evolution [30], and also provides a good opportunity to study protein-protein interactions in *Citrus*.

In this analysis, we predicted the sweet orange PPI network using ortholog-based and domain-based interaction methods, based on careful preparation and selection of reliable data resources, and then applied K-nearest neighbors (KNN) method to verify and filter the predicted PPIs and obtain a more confident network. The final PPI network in sweet orange, CitrusNet, contains 8,195 proteins and 124,491 interactions. This is the first report of a genome-wide PPI network in a woody plant. The quality of this PPIs network was carefully assessed. Furthermore, using RNA-seq data from four tissues, we analyzed important functional modules and obtained insights into the tissue-specific features of the sweet orange protein interactome.

## Results

### Topological properties of the PPI network

**Figure 1:**
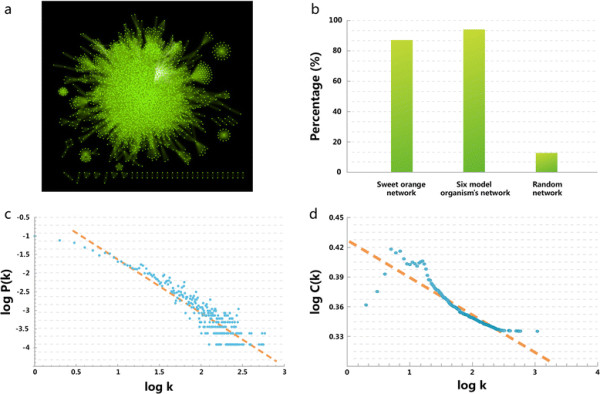
**Overview and general properties of CitrusNet. (a)** Overview of CitrusNet. **(b)** Ratio of KNN-verified PPIs in CitrusNet, six model organisms, and a random network. **(c)** Degree distribution of CitrusNet. **(d)** Cluster coefficient distribution of CitrusNet.

Based on a combination of ortholog- and domain-based prediction methods, we obtained 146,056 PPIs, and then assessed and filtered all the predicted PPIs by KNN. The final CitrusNet contained a total of 8,195 proteins and 124,491 interactions (Figure 1a). KNN verified 85.2% of the predicted PPIs, similar to the rate in six other model organisms (93.3%), and much higher than that of random PPIs (13.4%, Figure 1b), indicating that CitrusNet is highly accurate. When we used Cytoscape to visualize and analyze the topological properties of CitrusNet [31], 7,885 of the total 8,195 proteins were connected into a large interconnected sub-network. The topological properties indicated that CitrusNet was a scale-free network, *i.e.*, most nodes had low degrees of connection, whereas a few hub nodes had very high degrees of connection. In total, 602 proteins in CitrusNet had more than 100 connections. Table 1 lists the top ten hub nodes, including eight heat shock-related proteins, one polyubiquitin A protein, and one DNA-directed RNA polymerase II subunit protein. These data indicate that heat shock proteins play important roles in the citrus interactome. The degrees of the nodes in CitrusNet follows a power-law distribution fit by the regression equation *y* = 4885.8*x^-1.407^ (r^2^ = 0.851, P < 0.0001, Figure 1c), where *x* indicates degree and *y* indicates the number of nodes with a given degree.

**Table 1:** The top ten protein nodes with the highest degree in CitrusNet

**Protein name**	**Degree**	**Function**
Cs4g11150.3	1084	Polyubiquitin-A
Cs4g08220.1	582	Heat shock protein 81-3
Cs4g08220.3	582	Heat shock protein 81-3
Cs5g03150.1	559	Heat shock protein 83
Cs9g19220.1	557	Heat shock protein 83
Cs6g05890.1	543	Heat shock cognate 70 kDa protein 1
Cs7g29010.1	543	Heat shock cognate 70 kDa protein 2
Cs7g28940.1	535	Heat shock cognate 70 kDa protein
Cs8g18260.1	530	Heat shock cognate 70 kDa protein
Cs1g26080.1	499	DNA-directed RNA polymerase II subunit RPB1

Nodes with a high betweenness centrality (BC, which counts the fraction of shortest paths going through a node) are also very important for a network, because these nodes act as bottlenecks in the network even in the absence of hubs [32]. Table 2 lists the ten nodes with the largest BC in CitrusNet. Five of these nodes are also among the ten hub nodes with the highest degrees (Cs4g11150.3, Cs4g08220.1, Cs4g08220.3, Cs9g19220.1 and Cs5g03150.1); four of those are heat shock-related proteins, and the other is polyubiquitin A protein. Thus, all of these proteins play critical roles in the connection and communication among nodes in CitrusNet. Based on the combination of degree and BC analysis, we speculate that heat shock proteins are the most important proteins in the sweet orange PPI network.

**Table 2:** The top ten protein nodes with the highest BC in CitrusNet

**Protein name**	**BC**	**CC**	**Function**
Cs4g11150.3	0.146	0.448	Polyubiquitin-A
Cs1g26080.1	0.046	0.392	DNA-directed RNA polymerase II subunit RPB1
Cs7g30890.1	0.030	0.373	Clathrin heavy chain 1
Cs6g05890.1	0.023	0.418	Heat shock cognate 70 kDa protein 1
Cs7g29010.1	0.023	0.417	Heat shock cognate 70 kDa protein 2
Cs4g08220.1	0.021	0.413	Heat shock protein 81-3
Cs4g08220.3	0.021	0.413	Heat shock protein 81-3
Cs9g19220.1	0.021	0.411	Heat shock protein 83
Cs5g03150.1	0.019	0.412	Heat shock protein 83
Cs8g03630.1	0.017	0.385	Serine/threonine-protein kinase TOR

The clustering coefficient (CC, a measure of degree to which nodes in a graph tend to cluster together) of CitrusNet is 0.301, much higher than that of a random network with the same degree distribution (CC = 0.014). This feature indicated that CitrusNet is very intensive, and several sub-networks containing protein complexes or signaling pathways could be identified. The relationship between CC and degree in CitrusNet is shown in Figure 1d. From the distribution map, we observed that nodes with small degrees were intensively distributed, whereas nodes with large degrees were very sparse, further confirming the scale-free property of CitrusNet.

### Quality assessment of CitrusNet

**Figure 2:**
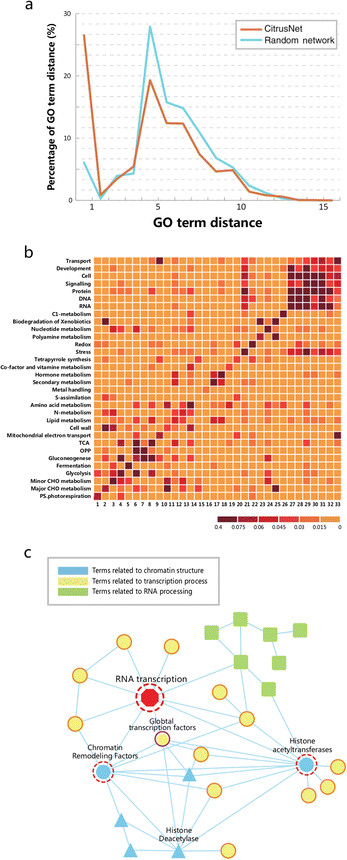
**GO term and Mapman validation of CitrusNet. (a)** Distance distribution of GO terms in CitrusNet and a random network. Orange line indicates CitrusNet prediction, and blue line represents the random network. **(b)** Heatmap of the correlation for different Mapman terms. Light yellow indicates that the two Mapman terms have relatively low correlation, and dark red indicates the two terms have relatively high correlation. **(c)** RNA transcription network based on Mapman annotations. Yellow nodes indicate terms related to transcription, blue nodes indicate terms related to chromatin structure, and grass green nodes indicate terms related to RNA processing.

Because no experimental PPIs are available in sweet orange, we employed gene ontology (GO) annotation analysis to evaluate CitrusNet. Because interacting proteins tend to have similar or related functions, a reliable PPI network should contain many more functionally related proteins than a random network. We performed GO analysis to compare the functional relationships among protein nodes in CitrusNet and random networks, and then obtained GO annotation from the GO database [33]. In total, 27,943 proteins in sweet orange had GO annotations, and 7,327 of these (constituting 109,409 interactions) were included in CitrusNet. For these comparisons, we calculated the shortest distance of GO terms for protein pairs in CitrusNet and five random networks. The number of proteins and protein pairs in random networks were the same as in CitrusNet, it is worth noting, however, that the protein pairs in random networks did not interact. Figure 2a shows that the shortest distance of GO terms in CitrusNet was much shorter than the average distance in five random networks.

Moreover, we analyzed many gene modules in CitrusNet to determine whether they contained known biological pathways in sweet orange. To this end, we used Mapman annotation, which represents biological functions with no redundancy between different terms [34]. We constructed a Mapman network for CitrusNet, and employed the clustering algorithm described in reference [35] to extract relevant modules. Figure 2b shows that the relevant Mapman terms were highly correlated, whereas terms with no functional relationships had low correlation. Detailed analysis of the RNA transcription sub-network illustrated that PPIs provide useful biological information (Figure 2c). In the RNA transcription sub-network, terms related to RNA transcription and global transcription factors exhibited complicated interactions (Figure 2c). Furthermore, terms related to chromatin remodeling factors, histone deacetylase activity, and histone acetyltransferase activity had many connections with the RNA transcription sub-network, suggesting that chromatin remodeling and histone acetylation/deacetylation might be closely related to the transcription process. The Mapman annotation network indicated that CitrusNet contained a considerable number of biological modules, and the clustering algorithm successfully grouped terms that were previously classified as multimeric complexes.

### Modular features of protein-interaction networks revealed by RNA-seq data analysis

**Figure 3:**
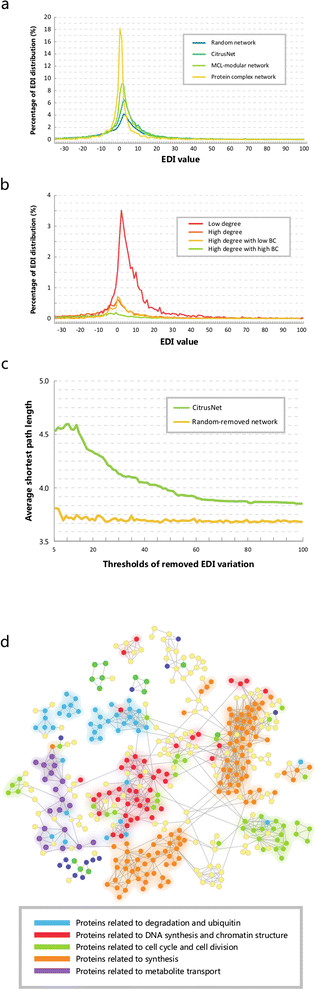
**The*EDI*distribution and modular features of CitrusNet. (a)***EDI* distributions for CitrusNet, random network, MCL-modular network, and protein-complex network. **(b)***EDI* distribution of CitrusNet based on topological properties. Red line represents low-degree nodes, orange line represents high-degree nodes, yellow line represents high-degree nodes with high BC, and grass green line represents high-degree nodes with low BC. Nodes with low-degree have the highest percentages of *EDI* values around zero. **(c)** Comparison of the average shortest path between CitrusNet and a random network, based on removed genes with different *EDI* variation values. **(d)** Modules in sub-networks of CitrusNet consisting of relatively highly expressed genes in four tissues. Node colors indicate different gene functions. Orange, synthesis; red, DNA synthesis and chromatin structure; blue, degradation and ubiquitination; purple, metabolite transport; grass green, cell cycle and cell division; yellow, other functions.

The relationship between gene expression and protein interactions has been comprehensively analyzed [36],[37]. In general, genes that encode interacting proteins are correlated at the level of mRNA expression [38]; therefore, correlations in gene expression should reflect the reliability of the predicted PPI network. Based on RNA-seq data from four sweet orange tissues (callus, leaf, flower, and fruit), we proposed an index, the expression difference of interacting protein pairs (*EDI*). Interacting genes with similar expression levels had *EDI* values closer to zero. We employed the Markov Clustering (MCL)-modular method [39] to extract relevant modules from CitrusNet. MCL is a clustering method for extracting relevant modules from the constructed interactome, and is superior to other clustering methods under most conditions [37]. We compared the *EDI* distributions of four CitrusNet-related networks, including CitrusNet, a random network, a MCL-modular network and a network inferred from yeast protein complexes [40]. In total, we obtained 159 protein complexes in sweet orange by using the best hits from sequence alignments with the yeast complexes (Additional file 1). Figure 3a shows the *EDI* distribution of these four types of networks. The protein complex network had the highest percentage of genes with *EDI* around zero, suggesting that protein complexes have the highest proportions of genes with similar expression levels. The MCL-modular network had a higher percentage of similarly expressed genes than CitrusNet, whereas the random network had the lowest percentage of *EDI* around zero, indicating that the MCL algorithm was highly effective at extracting relevant modules, and that the predicted CitrusNet was reliable. Currently, no protein complexes have been experimentally determined in sweet orange, therefore, the predicted 159 protein complexes in this analysis are very helpful for related studies.

We also analyzed the *EDI* distribution in CitrusNet based on its topological properties. First, we divided all genes in the network into two groups (genes with degree above ten were defined as high-degree genes, and other genes were defined as low-degree genes), and then divided the high-degree genes into two subgroups (high BC and low BC, respectively). Figure 3b illustrates that the percentage of low-degree genes with *EDI* around zero was much higher than the percentage of high-degree genes, because the high-degree genes had many interacting partners with different functions and expression levels, whereas low-degree genes had far fewer interacting partners whose functions and expression levels were more likely to be similar. This phenomenon reflects the ‘economical’ principle of gene interactions, *i.e.*, genes tend to interact with as few partners as possible to serve their biological functions. We also observed the same features for *EDI* and BC topological parameters. The percentage of high-degree/low-BC genes with *EDI* around zero was higher than that of high-degree/high-BC genes, suggesting that genes with low BC are likely to have similar expression levels.

In addition to studying the *EDI* distribution in different kinds of sub-networks, we also analyzed *EDI* variations among genes in the four tissues. We performed an *in silico* analysis to simulate the effect of removing specific nodes in the network. We defined different thresholds of *EDI*_*variation*_ values from 10 to 100, with a step size of 1. Based on these thresholds, we removed nodes with *EDI*_*variation*_ values higher than the thresholds in CitrusNet, and compared the average shortest path length after randomly removing the same number of nodes in CitrusNet. The shortest path length between two nodes reflects the overall network connectivity. In total, we removed 1658 nodes for *EDI*_*variation*_ = 10, and 117 nodes for *EDI*_*variation*_ = 100. The distributions of average shortest path lengths of these two types of interactome networks are shown in Figure 3c. The average shortest paths of *EDI*_*variation*_-removed networks were all longer than those of randomly-removed networks, indicating that nodes with high *EDI*_*variation*_ values were very important for network connectivity. Figure 3c also shows that nodes with *EDI*_*variation*_ values in the range 10–20 were crucial for the stability of the network, and that removing these nodes resulted in the largest shortest path lengths.

Finally, we analyzed the modular features of gene expression in CitrusNet. Figure 3d illustrates a sub-network comprising highly expressed genes in at least three tissues (Additional file 2). Genes with similar functions were clustered into modules. For example, genes related to protein degradation and ubiquitin (blue), DNA synthesis and chromatin structure (red), cell cycle and cell division (grass green), protein synthesis (orange), and metabolite transport (purple), are clustered into functional groups. The *EDI* values of genes in these modules were smaller than those of genes with different functions. Therefore, *EDI* value reflects the function relationships between interacting gene pairs. Based on this comprehensive analysis, we concluded that gene expression patterns in CitrusNet have modular features.

### Analysis of the plant hormone-related interaction network

**Figure 4:**
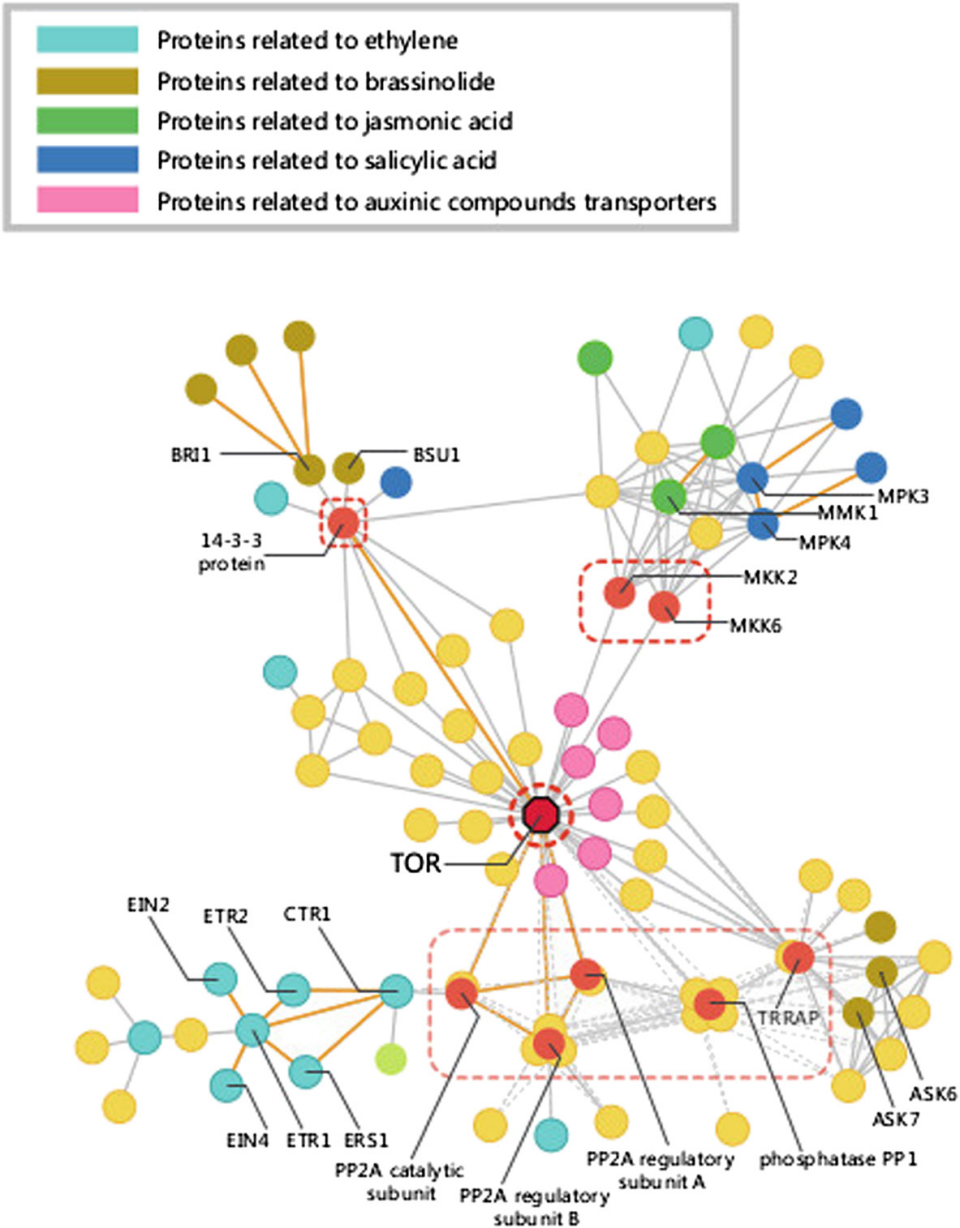
**Plant hormone sub-network.** Node colors indicates different plant hormone categories: Cyan, nodes indicate proteins related to ethylene; brown, proteins related to brassinolides; green, proteins related to jasmonic acid; deep blue, proteins related to salicylic acid; pink, proteins related to auxinic compounds transporters; yellow, proteins with other functions. Orange lines represent interactions supported by the literature.

Plant hormones such as ethylene, abscisic acid, auxins, gibberellins, cytokinins and brassinosteroids, play important roles in plant biology. To identify important hormone-signaling proteins in sweet orange, we constructed PPI sub-networks related to hormone-signaling pathways, based on information from the Arabidopsis Hormone Database (AHD2.0) [41]. These sub-networks were simplified by filtering proteins involved in basic biological process. In the hormone-signaling sub-network, proteins related to ethylene, jasmonic acid, salicylic acid, auxins, brassinosteroids and cytokinins formed a highly interconnected network, with target of rapamycin (TOR) as its center (Figure 4). In this sub-network, TOR had the highest BC, and therefore served as the hub protein. TOR is a conserved protein with similar functions in yeast, human and *Arabidopsis*[42],[43]. Although TOR has not been extensively studied in plants, experimental evidence in *Arabidopsis* has shown that this protein functions as a hub involved in the regulation of growth, stress resistance, and mRNA translation [44]. Proteins related to hormone signaling and hormone receptors do not interact with TOR directly, but through other connecting proteins. As shown in Figure 4, protein phosphatase 2A (PP2A) regulatory subunit and catalytic subunit both had direct interactions with TOR, and the catalytic subunit also interacted with the Raf-like protein kinase CTR1, which is a negative regulator of the ethylene response pathway in *Arabidopsis*[45]. Furthermore, jasmonic and salicylic acid signaling proteins had many interactions with each other, most of these interactions were mediated by mitogen-activated protein kinases (MPK4, MMK1), and many of them also interacted with mitogen-activated protein kinase-kinase MKK2 and MKK6, which directly interacted with TOR. Three different link paths connected brassinosteroid signaling proteins and TOR. Shaggy-related protein iota/kinase eta had similar interaction patterns, both interacted with TOR through protein phosphatase PP1 and phosphatidylinositol 3-/4- kinase (TRRAP). Brassinosteroid insensitive-1 (BRI1) and the downstream protein BSU1 connected with TOR through 14-3-3 protein. Six auxinic compound transporters directly interacted with TOR; therefore, we speculate that TOR regulates auxin signal transduction.

The links among different hormone signaling proteins were very complex, because several of the interacting proteins (PP2A, protein phosphatase PP1, TRRAP, 14-3-3 protein, and MAP kinase) were connected to a considerable degree. Many of the hormone-related proteins interacted with each other, indicating that various hormone-signaling pathways exchange information before affecting TOR (or vice versa). Although many plant hormone-related proteins were connected, TOR is undoubtedly the hub of hormone crosstalk. The central role of TOR implies that it integrates environmental information communicated by hormones, and uses this information to regulate plant growth.

## Discussion

In this study, we predicted the genome-wide PPI network of sweet orange, based on ortholog identification and domain-combination methods. Because there is no golden-standard PPI dataset in sweet orange, we employed a highly accurate KNN algorithm to filter the predicted interactions. The resultant PPI network, CitrusNet, contains 8,195 proteins with 124,491 interactions. We then employed GO and Mapman annotation to assess the predicted network. The shortest distance of GO terms in CitrusNet was much smaller than that of random networks, and the MapMan biology network demonstrated that gene modules in CitrusNet reflected known biological pathways in sweet orange. We also predicted 159 protein complexes in sweet orange using orthologs of the yeast protein complexes and employed them to assess CitrusNet; the results revealed that protein complexes had relatively tight connections. Together, these analyses confirm that CitrusNet is highly reliable.

We also analyzed the modular features of PPI networks based on the connections in CitrusNet. We proposed the *EDI* index to analyze the relationship between gene expression and protein interactions. The percentage of protein nodes with similar gene-expression levels in CitrusNet-derived sub-networks was much higher than those of random networks, especially in protein-complex and MCL-modular networks. Protein complexes are groups of two or more associated polypeptide chains, which are predicted to have tight network connections and to be encoded by genes that are expressed at similar levels. Analysis of the *EDI* distribution, based on topological properties, revealed that genes interacted with as few partners as possible (the ‘economical’ principle). Furthermore, *EDI* values around zero were negatively correlated with topological parameters such as degree and BC. In addition to analyzing *EDI* distributions, we also compared *EDI* variation among four tissues and found that genes with low *EDI*_*variation*_ values were important for the stability of CitrusNet.

Protein interactions play central roles in many different signal-transduction processes, including protein structure transformation, protein phosphorylation and dephosphorylation, and hormone signal processing. Many genes and biological processes regulated by hormone signals have been identified [46], and a growing number of studies have focused on the crosstalk among different hormone signals [47]–[49]. We constructed a PPI sub-network related to hormone-signaling proteins and observed that TOR serves as the central hub for hormone crosstalk. In general, TOR controls cell growth by regulating basic processes such as protein translation, RNA transcription, and protein degradation [50],[51]. Hormone signals pass through relay proteins, ultimately affecting TOR activity. Although TOR has been well studied in yeast, human, and *Arabidopsis*, its function in hormone-signaling pathways has not been previously investigated. Although we identified reliable connections between TOR and hormone-signaling proteins, the underlying regulatory patterns have not yet been elucidated. Many of the predicted plant hormone interactions in CitrusNet are supported by experimental evidence in other species [44],[50],[51]. For example, PP2A can down-regulate the activity of TOR [52], whereas the relationship between PP2A and another interacting protein CTR1 has not been previously reported. Considering that ethylene negatively regulates plant growth, we speculate that CTR1 can down-regulate the activity of PP2A catalytic subunit. Previous studies showed that 14-3-3 protein plays important roles in brassinosteroid signaling pathways [53]; however, the biological significance of the interaction between 14-3-3 protein and BRI1 remains unclear. Furthermore, 14-3-3 protein exerts a positive effect on TOR [54]; therefore, there may be a regulatory relationship between 14-3-3 protein and BRI1. The relationships between TOR and other plant hormone-related proteins have not been reported previously. Our analysis of the genome-wide PPI network illustrated the central role of TOR in hormone-signaling pathways. In the future, CitrusNet should provide further insights into the functional genomics and systems biology of sweet orange.

## Conclusions

In this analysis, we predicted the genome-wide PPI network of sweet orange using ortholog identification and domain-combination methods, and then employed a highly accurate KNN algorithm to filter the predicted interactions. The resultant PPI network contains 8,195 proteins and 124,491 interactions. We employed GO and Mapman annotation to assess the predicted network. We further predicted 159 protein complexes in sweet orange using orthologs of the yeast protein complexes and employed them to assess CitrusNet. We finally constructed a PPI sub-network related to hormone-signaling proteins, and found that TOR serves as the central hub for hormone crosstalk. CitrusNet provides a valuable resource for protein-protein interactions in sweet orange.

## Methods

### Data sources

Complete genomic sequence, annotation, and transcription data of sweet orange were obtained from the sweet orange genome annotation project [30]. RNA-seq data were obtained from four different tissues: flower, leaf, fruit, and callus. High-quality experimental PPI data from six reference model organisms, *A. thaliana, S. cerevisiae, C. elegans*, *D. melanogaster, H. sapiens* and *Mus musculus,* were collected from the public databases BIOGRID [21], IntAct [19], DIP [17], STRING [20], MINT [18], HPRD [22], and TAIR [55], To ensure the reliability of the data, we performed validation. Redundant sequences from different PPI databases were assigned to unique identification numbers using BLAST, with the following parameters: identity = 100% and coverage >99%. Final data are provided in Additional file 3.

### Methods for predicting PPIs

#### Ortholog identification

In a genome-wide statistical trend, orthologues are the most similar genes in different species, and are generally assumed to retain equivalent functions [56]. Therefore, if two orthologous proteins interact in one species, they are prone to interact in other species. We searched for orthologous proteins between sweet orange and each of the six reference organisms, using Inparanoid (standalone version 4.1) [57]. In this analysis, *A. thaliana* had a higher score than the other five organisms. First, all potential orthologs between sweet orange and each of the six reference genomes were identified, and then potential orthologs in different organisms were grouped together. Inparanoid scores were normalized in the range of 0–1, with a higher maximum threshold (4600) to retain conserved genes. If a protein corresponded to multiple orthologs in one reference genome, the orthologous protein with the highest score was retained. For any two proteins in sweet orange, if their orthologs in the six reference genomes had at least one experimentally verified interaction, the two proteins were predicted to interact. To rank candidate protein interactions, a normalized score was defined:

(1) $$\mathrm{Scor}e_{\mathrm{ortho}}=\sum_{i=1}^{6}\left(\mathrm{InScor}e_{A}\times \mathrm{InScor}e_{B}\right)$$

In equation (1), *InScore*_*A*_ and *InScore*_*B*_ indicated the normalized Inparanoid score for an interacting protein pair (*A* and *B*) in one reference organism. The final score for a protein pair was the sum of the scores in the six reference genomes. The threshold was set to 0.15, according to the distribution pattern of the score.

#### Domain-combination method

Domains, the fundamental functional units of proteins, participate in intermolecular interactions between related proteins. If the domains of two proteins are confidently known to interact, then the proteins themselves can be predicted to interact [11],[58],[59]. Consequently, PPIs can be inferred from their domain-domain interactions (DDIs). Han and colleagues considered that PPIs might be the consequences of groups of domains or multiple domain interactions; therefore, we applied their method to obtain accurate PPI networks based on DDIs [11]. We proposed the notion of domain combinations and domain-combination pair (dc-pair). To overcome the limitation of conventional domain-prediction methods, our model framework regarded dc-pairs as the basic units of PPIs. We set up the following probabilistic model to predict the interaction probability of protein pairs. Domains of sweet orange proteins were identified, using the Hmmer tool [60], to search against the Pfam database (including PfamA and PfamB) [61]. The threshold of Hmmer was set to 1e-3, and the domain alignment coverage was set to >0.6 for multi-domain proteins and >0.9 for single-domain proteins.

Suppose a protein pair *A* and *B* contained *m* and *n* domains, respectively. Then, Protein *A* and *B* have 2^*m*^-1 and 2^*n*^-1 possible domain combinations, and (2^*m*^-1) × (2^*n*^-1) dc-pairs would be obtained. The score for each dc-pair was defined as follows:

(2) $$\mathrm{Scor}e_{\mathrm{dc}-\mathrm{pair}}={\left[\left(2^{m}-1\right)\times \left(2^{n}-1\right)\right]}^{-1}$$

Using equation (2), all the dc-pair scores in the six model organisms were calculated. Considering that a dc-pair might appear in many different protein pairs with different scores, the highest score was selected for each dc-pair. After obtaining the scores of all the dc-pairs, we calculated the scores of potential interacting protein pairs in sweet orange. The final score for each protein pair (*A* and *B*) was defined as follows.

(3) $$\mathrm{Scor}e_{\mathrm{domain}}=\sum_{i=1}^{m}\sum_{j=1}^{n}\mathrm{Scor}e_{\mathrm{dc}-\mathrm{pair}}\times \frac{\mathrm{rated}-\mathrm{dc}-\mathrm{pairs}}{\mathrm{all}-\mathrm{dc}-\mathrm{pairs}}$$

In equation (3), *rated-dc-pairs* indicates the dc-pairs of protein pair *A* and *B* in the six reference organisms, and *all-dc-pairs* indicates all the dc-pairs of protein *A* and *B*. The final score in equation (3) denotes the protein pair (*A* and *B*) obtained from the domain-combination method. An appropriate threshold was set to obtain a credible result.

### Method for filtering and assessing PPI data

Because no experimental PPI dataset is available for sweet orange, we employed third-party cross-validation methods to filter and assess the predicted PPIs based on ortholog identification and domain-combination. K-nearest neighbor (KNN), a simple and accurate method to validate predicted PPIs, requires positive and negative training datasets [62]. In this analysis, the positive samples were experimentally determined PPIs in the six reference organisms, which were divided into five equal groups. The corresponding negative samples were randomly selected protein pairs from sweet orange, excluding the real or related protein pairs from positive samples (Pearson correlation >0.3). Each of the five groups had the same number of protein pairs in the positive and negative sample sets. The prediction results were checked by five independent training datasets, and predicted interactions supported by at least two datasets were kept. To compare the difference between PPI network and random network, this procedure was also performed for random networks.

### Methods for processing RNA-seq data

RNA-seq reads were generated by the Illumina and SOLiD platforms and aligned to the reference genome using TopHat (v1.2.0) and BioScope (v1.3) with default parameters [63]. Gene-expression levels for each RNA-seq library were calculated as reads per kilobase exons model per million mapped reads (RPKM) [64]. For hierarchical clustering analysis, we used clustering software Cluster 3.0 to perform complete linkage hierarchical clustering on genes, using uncentered Pearson’s correlation as the distance measure. Supposing that gene *A* interacted with *m* genes, we defined the expression difference of interacting genes (*EDI*) as follows.

(4) $$\mathrm{EDI}=\sum_{i=1}^{m}\left(\frac{\mathrm{RPK}M_{A}}{D_{A}}-\frac{\mathrm{RPK}M_{i}}{D_{i}}\right)$$

In equation (4), *RPKM*_*A*_ and *RPKM*_*i*_ indicate the RNA-seq expression values for gene *A* and its interacting partner *i*, and *D*_*A*_ and *D*_*i*_ indicate the degree of gene *A* and gene *i*. If gene *A* has *EDI* value close to zero, the expression levels of gene *A* and its interacting partner are similar.

After obtaining *EDI*, we calculated *EDI*_*variation*_ values for each gene in four tissues; callus, leaf, flower, and fruit. The *EDI*_*variation*_ value was defined in equation (5).

(5) $$\mathrm{ED}I_{\mathrm{var}\mathrm{iation}}=\mathrm{ED}I_{\mathrm{highest}}-\mathrm{ED}I_{\mathrm{lowest}}$$

*EDI*_*highest*_ and *EDI*_*lowest*_ represent the highest and lowest *EDI* of a gene in the four tissues, respectively. If a gene was not expressed in one tissue, we defined *EDI*_*lowest*_ as zero.

## Abbreviations

PPI: Protein-protein interaction

KNN: K-nearest neighbors

GO: Gene ontology

EDI: Expression difference of interacting genes

TOR: Target of rapamycin

AP-MS: Affinity purification mass spectrometry

Y2H: Yeast two-hybrid

BC: Betweenness centrality

CC: Clustering coefficient

DDIs: Domain-domain interactions

## Competing interests

The authors declare that they have no competing interests.

## Authors’ contributions

DYD and CJW collected the data, performed the analysis and wrote the draft manuscript. CLL and CYJ supervised the project and wrote the manuscript. GJ and CDJ set up the web server and helped to collect the data. LS helped to prepare the figures. XQ and DXX provided sequencing materials and revised the manuscript. All authors carefully read and approved the final manuscript.

## Additional files

## Supplementary Material

Additional file 1: The predicted 159 protein complexes in sweet orange, using best hits of sequence alignments from yeast complexes. Click here for file

Additional file 2: Genes highly expressed genes in at least three of the four tissues; callus, flower, fruit, and leaf. Click here for file

Additional file 3: High-quality PPIs for six model organisms obtained from various public databases. Click here for file
