# Supplementary material for: Prediction and functional analysis of the sweet orange protein-protein interaction network
Source: BMC Plant Biol. 2014 Aug 5;14:213. doi: 10.1186/s12870-014-0213-7 (PMC4236729; doi:10.1186/s12870-014-0213-7)
Supplement: Additional file 3: — High-quality PPIs for six model organisms obtained from various public databases. [file s12870-014-0213-7-S3.docx]

**Table S3.** High-quality PPIs for six model organisms obtained from different public databases

| **Species** | **Database** | **No. Proteins** | **No. PPIs** |
| --- | --- | --- | --- |
| ***Arabidopsis thaliana*** | TAIR, BIOGRID, INTACT, STRING | 5,675 | 27,365 |
| ***Homo sapiens*** | HPRD, BIOGRID, DIP, INTACT, MINT, STRING | 16,437 | 119,610 |
| ***Saccharomyces cerevisae*** | BIOGRID, DIP, INTACT, MINT, STRING | 8,308 | 189,030 |
| ***Musmusculus*** | BIOGRID, DIP, INTACT, STRING | 10,791 | 43,154 |
| ***Caenorhabditiselegans*** | BIOGRID, DIP, INTACT, MINT, STRING | 6,948 | 27,013 |
| ***Drosophila melanogaster*** | BIOGRID, DIP, INTACT, MINT, STRING | 11,133 | 57,653 |
